# Proceedings of the Gynæcological Society of Boston

**Published:** 1878-07

**Authors:** 


### PROCEEDINGS OF THE GYNÆCOLOGICAL SOCIETY OF BOSTON.

The ninety-first regular meeting of the Gynæcological Society of Boston was held, according to announcement in the *Journal*, at the Evans House, on the first Thursday in May, at three o'clock P. M. After the transaction of preliminary business, the doors were opened to such of the profession as had responded to the general invitation to attend. Among others present were Drs. Garratt, Gilman, Kimball, Marcy and Norris of Cambridgeport, Jones of Newton, and Wingate of Wellesley.

Dr. Cutter, active member of the society, proceeded to give a digest of fifty cases of uterine fibroids treated by electrolysis, conjointly by himself and Dr. Kimball.

The uterine fibroid had proved intractable under other methods of medical and surgical treatment, but results by electrolysis had exceeded expectation. Two ladies, who had been cured by this method, had kindly consented to be present, and were introduced to the society—their cases being given in detail.

Upon invitation of the chair, Dr. Garratt followed with brief remarks. The question of interest is, Does the electric fluid pass through the tissues? He formerly believed it did not; he now knows that it does. He had given a great deal of study to this question; an ordinary galvanometer will not solve the problem, but his own more perfected instrument had done so. The old idea that the human body resists the passage of the electric fluid is false; it is a better conductor than water; indeed, one of the best of conductors. He did not understand how the process was set up by which the tumor was removed, but that it accomplishes such removal there can be no doubt. Dr. Garratt farther observed, that when Dr. Cutter first called on him some years ago with the project, which had been so well developed and so completely justified by the paper just presented, he was skeptical; very willing that the experiment should be made, but he had no faith in any practical results. He was glad to testify that Dr. Cutter was right, and that he had gone farther in this department than he had himself.

Dr. Warner asked whether the electric fluid would as readily pass through dead as living flesh.

Dr. Garratt replied, no; experiments on the cadaver were not conclusive.

